# Peer Review of “COVID-19 National Football League (NFL) Injury Analysis: Follow-Up Study”

**DOI:** 10.2196/56040

**Published:** 2024-02-13

**Authors:** ­ Anonymous

**Keywords:** COVID-19, injury, prevalence, adaptation, sports medicine, follow-up, training, football, epidemiology, sport, athlete, athletic, injuries

*This is the peer-review report for “COVID-19 National Football League (NFL) Injury Analysis: Follow-Up Study.”*

## Round 1 Review

### General Comments

This paper [1] provides epidemiological data on injury incidences in the National Football League (NFL) before and after the COVID-19 lockdown. This paper has the potential to be clinically meaningful; however, it has major flaws that should be addressed before it is reconsidered.

### Specific Comments

#### Major Comments

1. The authors stated that “This is the first large scale opportunity to demonstrate the effects of these principles and how they are important to understanding injury epidemiology.” However, there are studies that have looked at the effect of COVID-19 on other sporting leagues, for example, Bundesliga, Major League Soccer (MLS), etc. All this has been published and should be cited.
2. Can the authors please confirm or comment on the potential accuracy of this open data? What validity checks were employed to demonstrate that these data are accurate?
3. How can the authors be sure that the increase in injuries was due to COVID-19?
4. What is meant by injuries? Soft tissue injuries or concussions? Perhaps an analysis on time-loss injuries would be more beneficial and add value.

## Abbreviations

MLS: Major League Soccer
NFL: National Football League
